# The Urinary Proteomic Profile Implicates Key Regulators for Urologic Chronic Pelvic Pain Syndrome (UCPPS): A MAPP Research Network Study

**DOI:** 10.1016/j.mcpro.2021.100176

**Published:** 2021-11-11

**Authors:** John W. Froehlich, Hsin-Hsaio Scott Wang, Tanya Logvinenko, Stephen Kostel, Shannon DiMartino, Adrie van Bokhoven, Marsha A. Moses, Richard S. Lee, MAPP Research Network

**Affiliations:** 1 Department of Urology, Boston Children's Hospital, Boston, Massachusetts, USA; 2 Department of Surgery, Harvard Medical School, Boston, Massachusetts, USA; 3 Department of Pathology, University of Colorado Anschutz Medical Campus, Aurora, Colorado, USA; 4 Vascular Biology Program, Boston Children's Hospital, Boston, Massachusetts, USA; 5 Department of Surgery, Boston Children's Hospital, Boston, Massachusetts, USA

**Keywords:** urine, UCPPS, bladder, TMT, inflammation, CD99, CD99 antigen, COL1A2, Collagen alpha-2(I) chain, COL3A1, Collagen alpha-1(III) chain, DEF4, Neutrophil defensin 4, ECM, extracellular matrix, FA, formic acid, GAGs, glycosaminoglycans, HC, healthy control, IGF2, insulin-like growth factor II, IL6, interleukin-6, IPA, ingenuity pathway analysis, MAPP, multidisciplinary approach to the study of chronic pelvic pain, MT1G, Metallothionein-1G, OSM, oncostatin M, PC, positive control, PIS, pooled internal standard, SULF2, extracellular sulfatase Sulf-2, TGFB1, transforming growth factor beta 1, TMT, tandem mass tag, TNFRSF12A, Tumor necrosis factor receptor superfamily member 12A, UCPPS, urinary chronic pelvic pain syndrome, XPNPEP2, Xaa-Pro aminopeptidase 2

## Abstract

Urologic chronic pelvic pain syndrome (UCPPS) is a condition of unknown etiology characterized by pelvic pain and urinary frequency and/or urgency. As the proximal fluid of this syndrome, urine is an ideal candidate sample matrix for an unbiased study of UCPPS. In this study, a large, discovery-phase, TMT-based quantitative urinary proteomics analysis of 244 participants was performed. The participants included patients with UCPPS (n = 82), healthy controls (HC) (n = 94), and disparate chronic pain diseases, termed positive controls (PC) (n = 68). Using training and testing cohorts, we identified and validated a small and distinct set of proteins that distinguished UCPPS from HC (n = 9) and UCPPS from PC (n = 3). The validated UCPPS: HC proteins were predominantly extracellular matrix/extracellular matrix modifying or immunomodulatory/host defense in nature. Significantly varying proteins in the UCPPS: HC comparison were overrepresented by the members of several dysregulated biological processes including decreased immune cell migration, decreased development of epithelial tissue, and increased bleeding. Comparison with the PC cohort enabled the evaluation of UCPPS-specific upstream regulators, contrasting UCPPS with other conditions that cause chronic pain. Specific to UCPPS were alterations in the predicted signaling of several upstream regulators, including alpha-catenin, interleukin-6, epidermal growth factor, and transforming growth factor beta 1, among others. These findings advance our knowledge of the etiology of UCPPS and inform potential future clinical translation into a diagnostic panel for UCPPS.

## Highlights

• The proteomics of urinary chronic pelvic pain syndrome (UCPPS) found altered pathways.
• Key changes among the extracellular matrix and inflammatory response proteins were found.
• Several of these pathways and proteins were exclusively altered in UCPPS.
• These findings may have diagnostic and/or therapeutic potential in the future.

## Graphical Abstract

**Figure:**
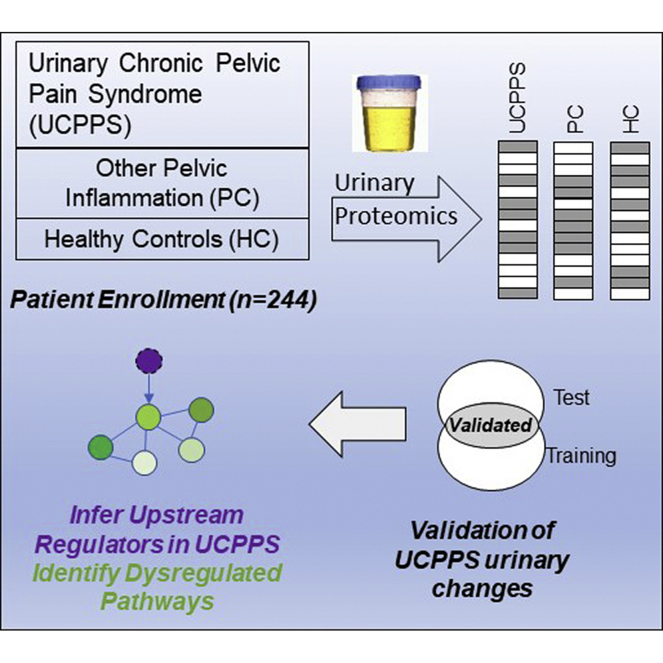

## In Brief

Urinary chronic pelvic pain syndrome (USPPS) is a poorly understood syndrome characterized by pelvic pain and urinary frequency and/or urgency. In this work, urinary proteomics was used to define the altered urinary proteome of UCPPS compared with healthy controls and unrelated pelvic pain diseases. The results describe the potential role of the altered extracellular matrix turnover and reflect an altered immune response. These findings may highlight novel treatment modalities and/or provide molecular targets for future diagnostic development.

Urologic chronic pelvic pain syndrome (UCPPS) comprised two urologic disorders, interstitial cystitis/bladder pain syndrome in males and females and chronic prostatitis/chronic pelvic pain syndrome in males. UCPPS presents with a high variability of symptoms. The estimates of prevalence vary widely, and diagnosis of UCPPS remains challenging. The syndrome is highly debilitating and effective treatments are sorely lacking (1). The etiology is largely unknown.

The National Institute of Diabetes and Digestive and Kidney Diseases established the Multidisciplinary Approach to the Study of Chronic Pelvic Pain (MAPP) Research Network to advance studies into the etiology, subgrouping and diagnosis of UCPPS, and treatment and management strategies. In this study, we used MAPP network samples to perform an unbiased quantitative proteomic analysis of urine from three cohorts: UCPPS patients, “positive controls” (PC) with chronic pain disease of a non-UCPPS origin, and healthy controls (HC). Although there are no discovery-phase urinary proteomic publications in UCPPS to date, hypothesis-driven approaches have previously implicated specific molecules in UCPPS. The published reports from our group have used ELISA to identify and validate urinary matrix metalloproteinases, neutrophil gelatinase-associated lipocalin, VEGF, and VEGFR1 in the context of UCPPS. These studies were able to discriminate between high and low levels of UCPPS severity and provide mechanistic insight into potential mechanisms underlying severity (2). Several of these molecules also correlated with longitudinal changes in symptom severity. Another hypothesis-driven study demonstrated higher responses of toll-like receptor (TLR) 2 and TLR4 stimulation in patient-derived peripheral blood mononuclear cells among females with UCPPS relative to healthy controls (3). The TLR4 response was also associated with lower pain threshold measured *via* an objective pain assay at the thumb, suggesting a higher systemic response to painful stimuli (4).

Although these hypothesis-driven studies have provided important insights into UCPPS, only large-scale, unbiased discovery experiments can enable assessment of multiple biological processes, networks, and regulators simultaneously. In this study, we assessed the quantitative urine protein profiles of 244 participants in the UCPPS, PC, and HC cohorts to define a urinary molecular signature associated with UCPPS. We further identified dysregulated networks and regulators of this syndrome. Confirmation of known affected processes was observed (*e.g.*, increased bleeding), as well as validation of several previously hypothesized etiological factors of UCPPS, such as compromised barrier function and immune signaling. The key differences between the male-specific and female-specific UCPPS proteomes are also discussed. Collectively, these findings will be important in guiding future hypothesis-driven studies of key proteins affecting the UCPPS phenotype. Furthermore, this study identifies the potential targets for treatment by identifying key upstream regulators that are predicted to be specific drivers of the urinary proteomic changes in UCPPS.

## Experimental Procedures

### Cohort Generation

The MAPP Research Network was formed to define UCPPS phenotypes, etiologies, and outcomes. All the participants provided written informed consent following an IRB-approved protocol. The studies in this work abide by the Declaration of Helsinki principles. The samples from HC, UCPPS, and PC consisting of individuals with distinct chronic pain conditions were included in this study. Diagnoses for PC included fibromyalgia, chronic fatigue syndrome, and irritable bowel syndrome. Demographics and patient reported scores with regards to pain and urinary symptoms were obtained from the MAPP network, as previously described (5, 6). The details for individual samples are presented in supplemental data 1. The urine samples (244 in total) were obtained from multiple study centers and coordinated by the MAPP Network Tissue Analysis and Technology Core *via* a centralized IRB-approved protocol. The samples were shipped from Tissue Analysis and Technology Core on dry ice and immediately transferred to −80 °C storage before sample preparation. Phenotypic data and other aspects of sample information were coordinated *via* the MAPP network Data Coordinating Center. Patient demographics and the collected reported clinical data are summarized in Table 1. The pain and urinary severity scores were determined by the validated Genitourinary Pain Index questionnaire (7).

**Table 1:** Cohort demographics

Cohort	N	# Females (%)	Median age (IQR)	Median pain (IQR)	Median urinary (IQR)
UCPPS	82	43 (52.4)	42.3 (31.0–53.0)	12.0 (7.0–20.8)	10.0 (5.0–18.0)
HC	94	55 (58.5)	40.4 (28.2–51.4)	0.0 (0.0–0.0)	1.0 (0.0–2.8)
PC	68	52 (76.5)	44.0 (30.9–53.5)	0.0 (0.0–1.5)	3.0 (1.8–5.3)

Abbreviations: HC, Healthy Control; PC, Positive Controls; UCPPS, Urologic Chronic Pelvic Pain Syndrome.

UCPPS was characterized by higher pain and urinary severity scores than the other cohorts. Full cohort demographics are presented in supplemental data 1.

### Sample Preparation

The samples were desalted, reduced, and alkylated with iodoacetic acid in a 10 kDa molecular weight cutoff filter (Amicon Ultra) using a previously published, spin-filter based protocol (8, 9, 10). Proteins were harvested from the filters and quantified by the BCA assay (Thermo). A constant 20 μg of each sample were aliquoted, dried, and digested with trypsin overnight in 50 mM TEAB buffer. After digestion, each sample was labeled with TMT6plex reagent according to manufacturer's instructions, quenched, and mixed into TMT labeling groups. Each labeling group consisted of five study samples and one pooled internal standard (PIS), which was generated by pooling urine from six healthy adult males and six healthy adult females. Digestion was performed using trypsin at a 1:40 enzyme:substrate ratio for 16 h. After digestion, the samples were acidified with formic acid (FA) and were dried to dryness using a speed vac (Thermo). The peptides were cleaned by reverse-phase solid-phase extraction (Oasis HLB cartridges, Waters). After conditioning, equilibrating, and loading by standard approaches (10, 11), the samples were washed 4 times with 0.1% FA and eluted using 50% acetonitrile in water with 0.1% FA. The eluates were dried to dryness.

### Mass Spectrometry

TMT-groups were reconstituted in MS loading buffer composed of 5% FA and 5% acetonitrile. The samples (0.75 μg) were loaded onto a Thermo Q Exactive HF-X MS using an Easy1200 nLC at a flow rate of 1 μl/min. A chip system (picoChip, New Objective) was used to trap and elute the peptides. The stationary phase was Reprosil PUR 3um 120 Å, 3 microns, 25 cm. A 60 min gradient from 5% buffer B (0.1% FA in acetonitrile) to 30% B (in 52 min) followed by a gradient from 30% B to 45% B in 8 min. Buffer A was 0.1% FA in water and buffer B was 0.1% FA in 80% acetonitrile, and the flow rate was 500 nl/min.

MS^1^ settings were as follows: range 350 to 1450 *m/z*; resolution 120,000; Automatic Gain Control target 3e6; 50 ms maximum ion accumulation time; one scan range (single scan); and profile data were obtained. Tandem MS^2^ scans were obtained as follows: A top 16 method was used with a fixed first mass of 100 *m/z* with dynamic higher mass range; isolation window was 0.8 *m/z*; AGC target of 1e5; 80 ms maximum ion accumulation time; collision energy of 32 normalized collision energy; and an underfill ratio of 10.0%. Dynamic exclusion was set to 15 s, and the precursor charge states 2 to 5 were permitted.

### Database Search Strategy

The RAW MS data were loaded directly into Proteome Discoverer (version 2.2). The spectra were calibrated using the spectral recalibration node within Proteome Discoverer and searched against the full reviewed uniprot *homo sapiens* database July 2019 release (20,416 sequences), using Byonic (v2.16.11). The search parameters for spectral recalibration were as follows: precursor tolerance 10 ppm; fragment tolerance 0.05 Da; trypsin fully specific digestion; 0 missed cleavages; fixed carbamidomethyl cysteine residues, fixed TMT6plex (peptide N-terminus and lysine). The full reviewed -UniProt *homo sapiens* database July 2019 release (20,416 sequences) with the addition of Byonic common contaminants was used for the final recalibrated database search. Byonic search parameters were as follows: precursor tolerance 8 ppm; fragment tolerance 15 ppm; trypsin semi-specific protease (C-term); one missed cleavage; fixed carbamidomethyl cysteine residues, TMT6plex (peptide N-terminus and lysine); and oxidation of methionine (common 2), deamidation of asparagine (common 1), and (de)TMT6plex (rare 1) as variable modifications. Byonic default recommendations were employed to generate 2D FDR values. The spectra had an automatic score cut applied on the peptide level, and the proteins were filtered at a 1% FDR level. To be used for quantification, TMT reporter ions required a 10mmu tolerance, an average reporter ion signal-to-noise ratio of at least 3, a 5% 1D FDR level, and a maximum of 50% precursor coisolation intensity was permitted. Unique + razor peptides were permitted for quantification.

### Experimental Design and Statistical Rationale

**Fig. 1:**
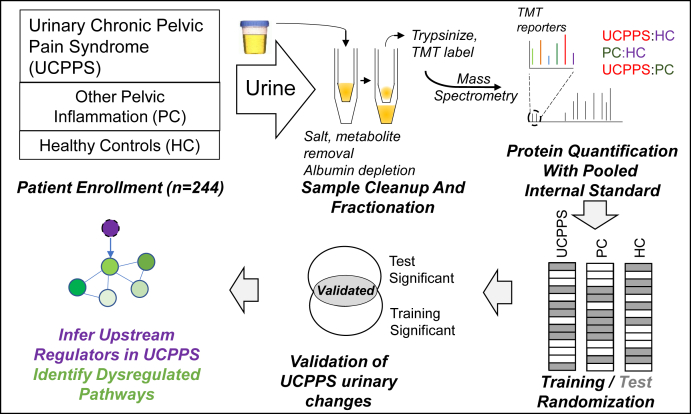
**Experimental approach. An overview of the experimental approach used in this study.** Urine from UCPPS, PC, and HC cohorts (244 in total) were processed and labeled with TMT for quantification. A pooled internal standard was added to each labeling group. The individual samples were randomly assigned to training or test groups (1:1 split) to define validated quantitatively altered proteins. The proteins comprising the union of the training and test set significant proteins were considered validated. Dysregulated pathways and upstream regulators of the empirical urinary changes were determined using the full set of data. HC, healthy controls; PC, positive control; TMT, tandem mass tag; UCPPS, urinary chronic pelvic pain syndrome.

Study experimental design is shown in Figure 1. The samples were labeled with TMT individually. To facilitate the use of TMT quantification with large cohorts, each TMT6plex label group included one PIS and five study samples. For each individual sample, the per-protein ratio relative to the corresponding PIS TMT channel was calculated by averaging the reporter ion intensities of all individual peptide identifications for a single protein. The full set of data are presented in supplemental data 2-7. The technical replicates were performed and displayed a high level of correlation (R = 0.92, supplemental data 1). Each study subject was randomly placed, without duplication or overlaps, into fully independent training or test sets to identify a set of validated statistically significant markers of UCPPS: PC and UCPPS: HC differences. The training-test set assignments maintained an equal split of UCPPS, PC, and HC between the training and test sets and are presented in supplemental data 1. To satisfy the requirements for validation, a protein was required to have (1) a 0.05 *p* value (parametric, two tailed tests) in each of the training and test sets and (2) a consistent direction of fold-change in both training and test sets.

To most effectively leverage the large cohort proteomic data for network and upstream regulator analyses, *p*-values were generated for individual proteins using the complete dataset, ignoring the training-test splits that were used for cohort-wide validation experiments. To generate any of the comparative ratios in this study, a minimum of the measurements of ten subject's were required. Each individual subject's protein were as follows: PIS log_2_ value was used for generation of p-values. Proteins with a *p*-value of 0.05 or lower were used for all network analysis, upstream regulator, and other analyses using ingenuity pathway analysis (IPA), described in Kramer *et al.* (12). Quantitative ratios were calculated by averaging the individual subjects' protein: PIS ratios for the two comparisons. Subsections of the data that were analyzed included cohort-wide comparisons, sex-specific UCPPS:HC ratios, and binary groups of UCPPS symptom severity (high/low). For all IPA analyses, default settings were used in the October 2020 IPA release. Unless otherwise mentioned, IPA-generated z-scores have an absolute value (|z|) of two or greater.

## Results

### Identifying Validated UCPPS: PC and UCPPS: HC Urinary Proteins *via* Training-Test Procedure

**Fig. 2:**
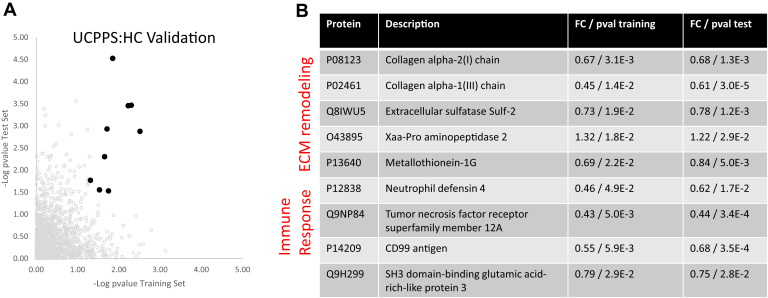
**Validation of quantitatively altered UCPPS:HC proteins.** Validated proteins in the UCPPS: HC comparison are plotted in (*A*) (*filled black circles*) and tabulated at *right* (*B*). The proteins that did not satisfy a p value < 0.05 in both the training and test sets are plotted in *empty gray circles*. Functionally, the set of validated proteins largely reflect ECM components/remodeling and immune responses. ECM, extracellular matrix; HC, healthy controls; UCPPS, urinary chronic pelvic pain syndrome.

The study design (schematically shown in Fig. 1) yielded *p* values of validated proteins for the UCPPS: HC training and the test-sets are plotted in Figure 2*A* (black circles). All nonvalidated proteins are visualized in gray circles. A total of nine proteins satisfied the UCPPS: HC validation criteria and they are tabulated in Figure 2*B*. Several of these are components of the extracellular matrix (ECM), or ECM-altering enzymes, including Collagen alpha-2(I) chain (COL1A2), Collagen alpha-1(III) chain (COL3A1), extracellular sulfatase Sulf-2 (SULF2), Xaa-Pro aminopeptidase 2 (XPNPEP2), and Metallothionein-1G (MT1G). Three other proteins influence immune responses: Tumor necrosis factor receptor superfamily member 12A (TNFRSF12A), CD99 antigen (CD99), and Neutrophil defensin 4 (DEF4). A total of three proteins were validated in the UCPPS: PC comparison using identical criteria. The three validated UCPPS: PC proteins were COL3A1 chain, retinoic acid receptor RXR-beta, and insulin-like growth factor II (IGF2).

### Global UCPPS: HC Quantitative Differences, and Upstream Regulators

**Fig. 3:**
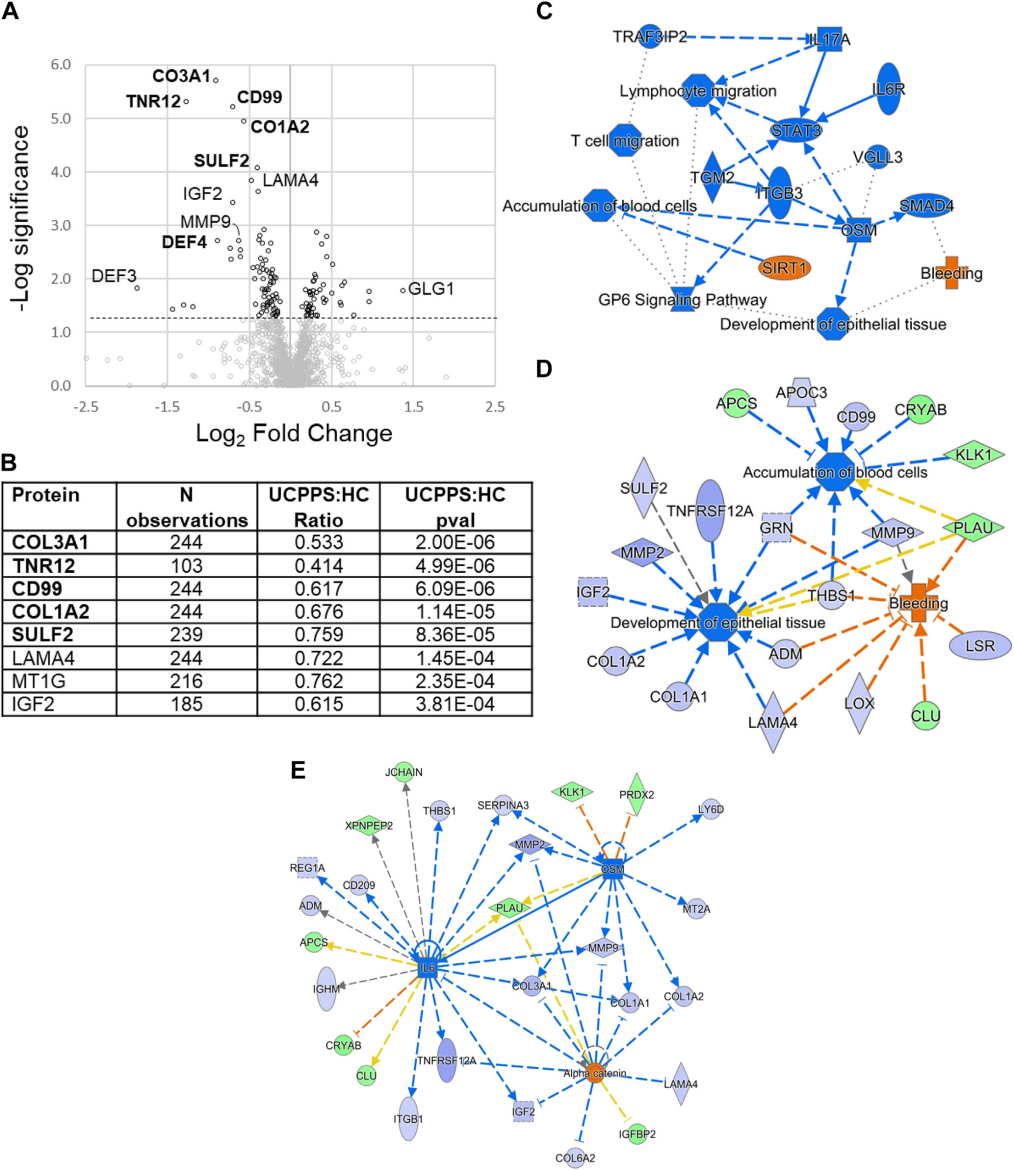
**UCPPS: HC proteins, regulators, networks.***A*, all quantitatively varying proteins are shown in *black circles*, and nonvarying proteins are *gray*. *B*, notable proteins are labeled and tabulated below, along with the count of quantitation of subjects. Train-test set validated proteins are *bolded*. *C*, a graphical overview of selected UCPPS:HC altered regulators and biological processes is shown. For *C–E*, *dashed lines* indicate an indirect relationship, and *solid lines* indicate a direct relationship. The *standard arrowheads* indicate increases/activation and *stopped lines* indicate decreases/inhibition. The decreased nodes are indicated by *light blue* (measured) or *dark blue* (predicted) to decrease, whereas the increased nodes are indicated by *orange* (predicted) or *green* (measured). *Blue*- and *orange-colored lines* reflect agreement between measurement and predicted values. Functionally, the altered levels of empirically measured urinary proteins indicate increased bleeding and decreased accumulation of blood cells and the development of epithelial cells (Fig. 3*D*). Several of the urinary protein alterations in UCPPS could be caused by dysregulation of three upstream regulators: IL6, OSM, and alpha catenin (Fig. 3*E*). HC, healthy controls; IL6, interleukin-6; OSM, oncostatin M; UCPPS, urinary chronic pelvic pain syndrome.

Having identified several validated UCPPS: PC and UCPPS: HC proteins of interest, we proceeded to conduct a comparative study of altered biological processes and functional categories using the full set of UCPPS, PC, and HC quantitative data. A volcano plot noting several proteins of interest for the full UCPPS: HC comparison is shown in Figure 3*A*, with results tabulated in Figure 3*B*. Every validated protein was significant in the full merged dataset. The bolded proteins were validated in the training-test split detailed above. Additional proteins of note include Laminin subunit alpha 4 (LAMA4), IGF2, matrix metallopeptidase 9 (MMP9), defensin 3 (DEF3), and Golgi glycoprotein 1 (GLG1). Several of these narrowly missed inclusion in the validated lists above. In total, 145 proteins satisfied the described criteria for IPA analysis for UCPPS: HC.

Figure 3*C* shows a high-level overview of altered master regulators and biological functions in the global UCPPS: HC dataset. This was generated from empirical measurements of differential proteins using IPA. The blue color denotes a predicted decrease of a particular node, whereas orange denotes an increase. Each node represents either biological functions (*e.g.*, accumulation of blood cells) or predicted upstream regulators (*e.g.*, SIRT1) of the identified changes. The IPA *p* values reflect enrichment of a set of proteins/genes in a dataset that are related to a particular function or regulator, whereas z scores predict increases and decreases in activity. Each node shown in Figure 3*C* had a |z| score of greater than 2. Figure 3*D* shows the influence of the empirically measured UCPPS: HC proteins on three selected UCPPS-relevant biological functions: bleeding, accumulation of blood cells, and development of epithelial tissue. A predicted increase in bleeding in UCPPS is driven by increases of PLAU and CLU and decreases of seven urinary proteins (MMP9, LSR, LOX, THBS1, ADM, LAMA4, and GRN). Decreased accumulation of blood cells is driven by a few of the above listed proteins along with CD99 and APOC3 in addition to increased KLK1, CRYAB, and APCS. The decreased COL1A2, SULF2, and IGF2 among others in the UCPPS cohort contribute to a decrease in the predicted level of the development of epithelial tissue term.

An IPA upstream regulator analysis predicted several putative upstream regulators (summarized in supplemental data 1). A manually generated network of three putative upstream regulators is shown in Figure 3*E*, along with the measured urinary proteins. Predicted activation of alpha catenin (shown in orange) is a result of decreases in several ECM components (LAMA4, COL1A1, COL1A2, and COL6A2) and decreases in secreted proteases (MMP9 and MMP2). In concert, this suggests a decreased amount of ECM turnover detected in the urine of UCPPS relative to HC, potentially because of increased alpha catenin signaling. Many of the same urinary proteins are affected by the predicted decrease of interleukin-6 (IL6) and oncostatin M (OSM) signaling. In addition, the hypothesized alterations of both alpha catenin and OSM cause a decrease in IL6, forming a connected signaling network and potential “feed-forward” dynamic (13, 14).

### Sex-Specific Differences in UCPPS: HC

HC urine samples contained several gender-enriched proteins. These included both male- and female-enriched proteins, and they are listed in supplemental data 1. Male-specific and female-specific UCPPS:HC ratios were generated separately to investigate potential differences in the networks of quantitatively varying proteins and upstream regulators in males and females. Comparing these datasets, the differences between male and female UCPPS: HC changes are clearly present at the protein, regulator, and network levels. In males, the most significant UCPPS: HC proteins are two collagens, CO3A1 (FC = 0.35, *p* = 7.7E-6) and CO1A2 (FC = 0.54, *p* = 2.5E-5). In females, although these collagens were significantly altered, the most significant UCPPS: HC protein was the functionally distinct Golgi apparatus protein 1 (GSLG1, FC = 1.62, *p* = 1.5E-3). In total, males had more statistically significant proteins and more upstream regulators satisfying a *p*-value of 1E-4 (152 *versus* 41). To identify sex-specific networks and regulators, a comparison analysis of UCPPS:HC proteins specific to males and UCPPS:HC specific to females was performed in IPA, identifying differences in several upstream regulators. The full set of *p* values and z scores is summarized in supplemental data 1.

**Fig. 4:**
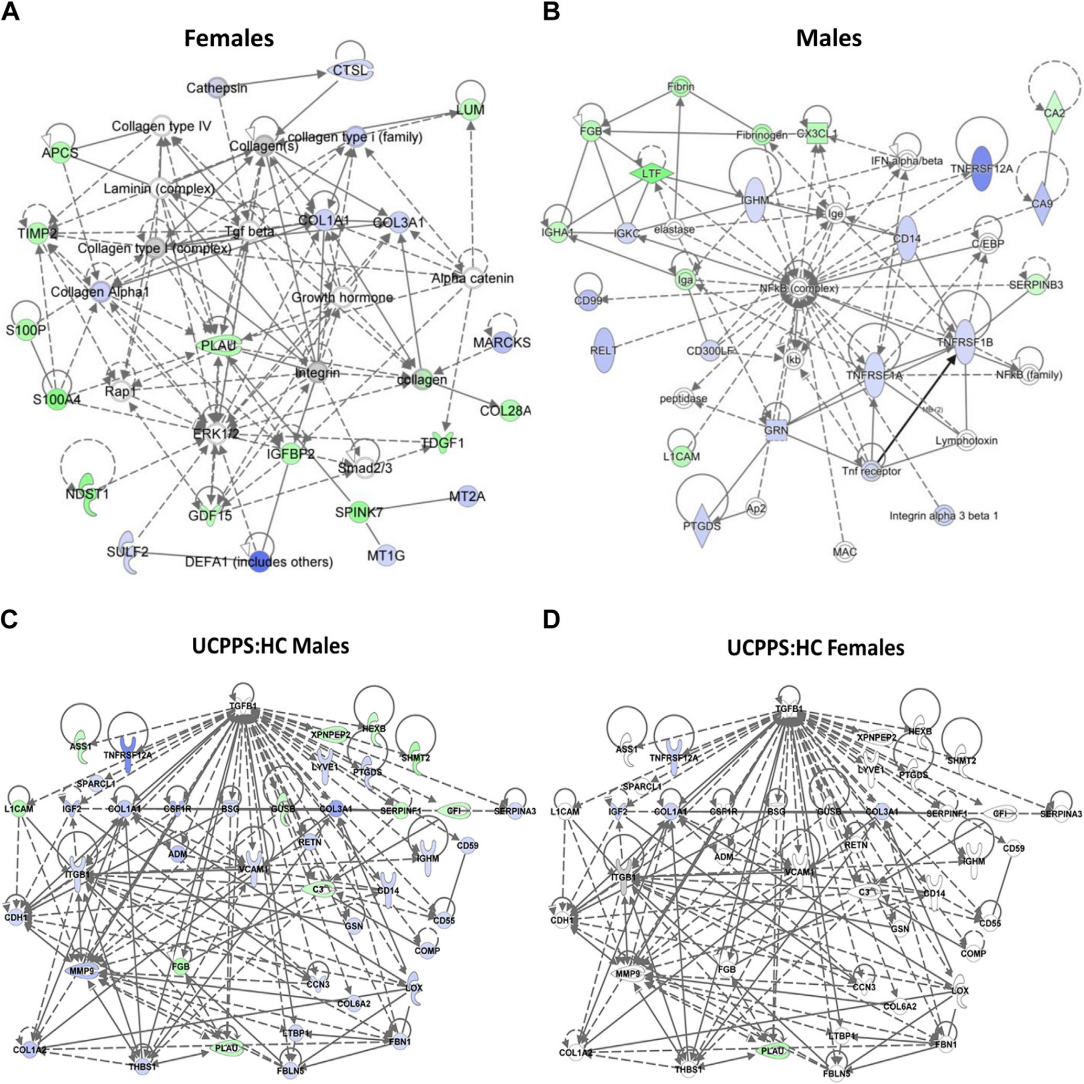
**Sex-specific UCPPS: HC effects.** Quantitatively UCPPS urinary proteomes are highly distinct between females and males. This is readily apparent by comparing the top networks of quantitatively altered proteins in females (*A*) and males (*B*). The top female UCPPS:HC network is characterized by ECM components, whereas the male network is dominated by immunomodulatory proteins. Clear differences are also observed on the level of upstream regulators. The upstream regulator is TGFB1 is shown in (*C*) and (*D*), along with statistically significant altered urinary proteins. Males (*C*) have a clear enrichment of dysregulated proteins downstream of TGFB1, whereas the females do not. *Green entries* are increased, *blue* are decreased, and *gray* are nonsignificant in the corresponding sex-specific UCPPS: HC comparisons. ECM, extracellular matrix; HC, healthy controls; TGFB1, transforming growth factor beta 1; UCPPS, urinary chronic pelvic pain syndrome.

As one example of the contrasting quantitative profiles present in the sex-specific comparisons, the top networks for males and females are shown in Figure 4, *A* and *B*. Contrasting biological functions are apparent in the two networks. The male network is functionally associated with immune signaling and contains the NFkB complex, TNF, and interferon, whereas the top female network contains ECM components: collagens, integrin, and alpha catenin. In concert, these sex-linked differences in the urinary proteome of UCPPS reflect large differences that may have functional or etiological significance in gender-specific UCPPS. As another example of gender differences in UCPPS: HC comparisons, a visualization of transforming growth factor beta 1 (TGFB1)-related urinary proteins is shown in Figure 4, *C* and *D*. In both the male and the female datasets, TGFB1 emerges as a highly significant upstream regulator (*p*-value_males = 1.2E-14 and *p*-value_females = 1.7E-7), although with widely differing z-scores (males = −1.4 and females = 0.4), reflecting a more inconsistent quantitative profile of TGFB1-related proteins in females.

### UCPPS: PC Proteins and Networks

**Fig. 5:**
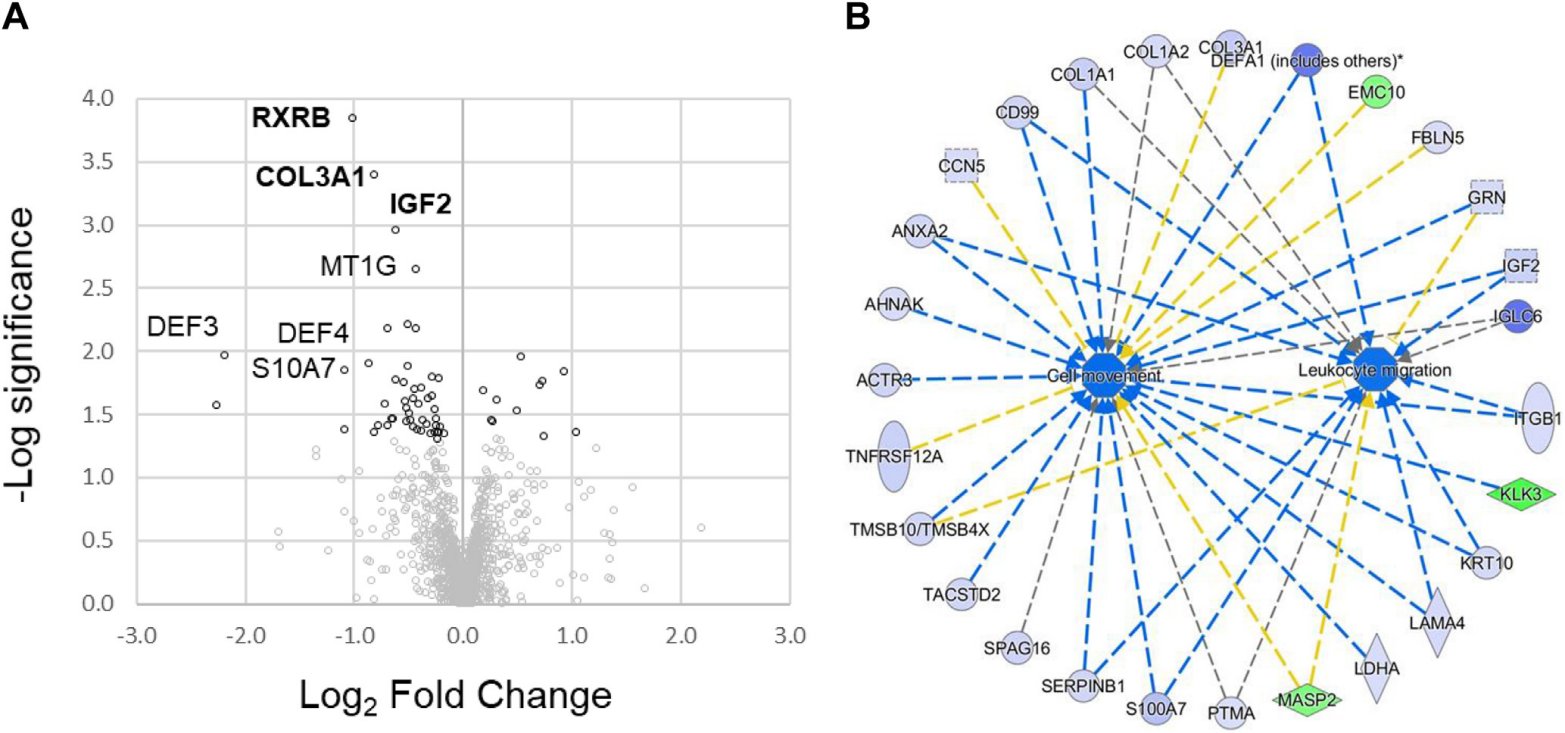
**UCPPS: PC proteins and functional alterations.** UCPPS: PC proteins and networks. In (*A*), protein level -log_10_ significance and log_2_ fold-change values are plotted. *Bold* proteins were validated in a training-test comparison. In (*B*), decreases in cell movement and leukocyte migration are predicted by the empirical changes in urinary proteins. *Green entries* are increased and *blue* are decreased. Predicted activation/inhibition relationships for each of the functional categories are shown by edges connecting the individual protein nodes to the functional categories. The *blue* colored *nodes* predict decreases in the functional categories and *yellow* predict activation. Log_2_ Fold Change. PC, positive controls; UCPPS, urinary chronic pelvic pain syndrome.

The PC cohort used in this study represents distinct chronic pain diseases, including chronic fatigue syndrome, fibromyalgia, and irritable bowel syndrome. These diseases share some characteristics with UCPPS, such as chronic abdominal pain and periodic flares in some patients and fatigue. UCPPS is notably distinct from each on account of the presence of substantial urinary symptoms, in addition to other symptoms. Although the etiologies for each of these diseases have not been fully determined, research has implicated improper immune responses for each. Differences in the quantitative protein profile of the UCPPS cohort relative to PC may reveal key proteins that contribute to the unique bladder presentation of UCPPS rather than the shared chronic pain aspects of UCPPS and PC. Three validated proteins, COL3A1, Retinoic acid receptor RXR-beta (RXRB), and IGF2 were identified in the UCPPS: PC comparison (Fig. 5*A*, bolded). Each of these can have multiple functional consequences relevant to UCPPS pathology. Retinoic acid influences myriad immune responses, including cytokine production, lymphocyte activation, and induction of regulatory t-cells. IGF2 and COL3A1 similarly influence wide-ranging biological functions, depending on the context. Given the multimeric functions of these validated proteins, we reasoned that considering the complete set of altered set of varying UCPPS: PC, urinary proteins might shed light on dysregulated pathways. We therefore used IPA to determine that these sets were enriched in proteins reflecting decreased cell movement and leukocyte migration. These are presented in Figure 5*B*.

### Comparative Analysis of Upstream Regulators Across Cohorts

**Fig. 6:**
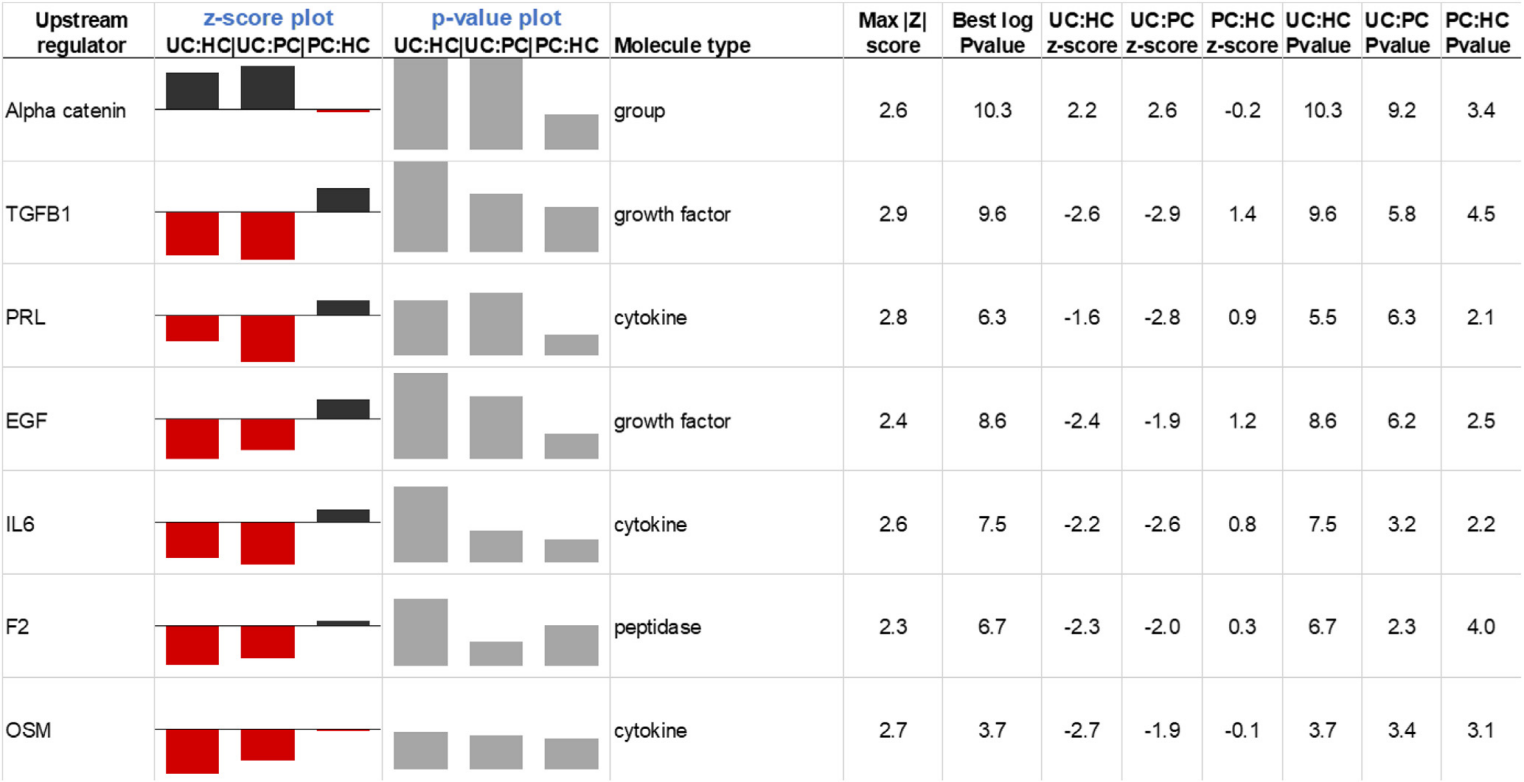
**A comparative analysis of shared upstream regulators in UCPPS, PC, and HC.** As described in text, seven upstream regulators satisfied the criteria to be considered “UCPPS-specific” in this study. They are alpha-catenin, TGFB1, PRL, EGF, IL6, F2, and OSM. The z-scores and −log *p* values for each regulator are shown graphically and listed at *right*. EGF, epidermal growth factor; F2, prothrombin; HC, healthy controls; IL6, interleukin-6; OSM, oncostatin M; PC, positive controls; PRL, prolactin; TGFB1, transforming growth factor beta 1; UCPPS, urinary chronic pelvic pain syndrome.

Although many studies compare a disease and a control condition, the inclusion of a distinct disease cohort (PC) in this study has enabled an integrated comparison of all three cohorts to identify UCPPS-specific regulators that may differentiate UCPPS as a specific chronic condition from other PC-based diagnoses. An upstream regulator was denoted “UCPPS-specific” for this study if it satisfied four criteria: 1. Statistically significant at the 1E-3 level in both UCPPS: HC and UCPPS: PC comparisons; 2. Had a consistent increase or decrease in the UCPPS:HC and UCPPS: PC z-scores; 3. Had a |z|>2 in one of the UCPPS: HC or UCPPS: PC comparisons; and 4. Did not have a |z|>2 in the PC:HC comparison. The set of seven upstream regulators that satisfied these conditions is shown in Figure 6. Several of these emerged previously in the UCPPS: HC global or sex-specific comparisons, including IL6, OSM, TGFB1, and alpha catenin, highlighting the UCPPS-specific nature of these findings. In addition, epidermal growth factor, prolactin, and prothrombin are predicted to have decreased signaling that is specific to UCPPS. Intriguingly, despite shared phenotypic characteristics of inflammation and pain between PC and UCPPS, urinary levels of the cytokines TGF-beta 1 and IL-6 are specific to the UCPPS cohort and are therefore similar in the UCPPS: PC and UCPPS:HC comparisons. We hypothesize that despite a presumed elevated inflammatory state, overall, the PC cohort does not demonstrate these changes in urine to the same extent as the UCPPS cohort, potentially because of the bladder-focused nature of UCPPS compared with PC.

## Discussion

UCPPS is an idiopathic condition characterized by pelvic pain and urinary urgency, frequency, or both. The primary objective of this study was to identify and validate putative biomarker proteins characteristic of UCPPS, by comparison with the HC and PC cohorts. To achieve this, we randomly divided each of the UCPPS, HC, and PC patient cohorts into two sex-balanced halves to form training and test sets. We obtained a small, distinct, validated set of nine UCPPS: HC proteins and three UCPPS: PC proteins. Functionally, these included components of the ECM, ECM-processing enzymes, and proteins that influence immune responses, which are each highly relevant biological processes in UCPPS.

Validated UCPPS: HC proteins included COL3A1 and COL1A2, two collagens that were each decreased in UCPPS. In the healthy bladder, collagens are important structural elements, contributing to the filling and compliance of the bladder (15). Collagens are abundant members of the healthy urinary proteome (9) and likely reflect a healthy level turnover of bladder ECM. UCPPS is an inflammatory syndrome, and loss of urinary collagen could reflect a wound healing process or disruption of the healthy bladder ECM. The additional ECM changes are implied by an increase in the XPNPEP2 protease and decreases in the MT1G protease and the heparin sulfate-modifying sulfatase SULF2. SULF2 removes the 6-O sulfate from Heparin sulfate, releasing chelated growth factors and chemokines, and affecting subsequent signaling (16). Glycosaminoglycans (GAGs) form protective barriers in the bladder and influence inflammation (17), adhesion, mitogenesis, leukocyte adhesion, and other biological functions (18). Changes to GAGs have been hypothesized to have a causative role in UCPPS (19), have been proposed as biomarkers of UCPPS (20), and intravesical treatment with GAGs can be therapeutic (21). In summary, prior studies of UCPPS bladder tissue are not numerous, however there is evidence for altered ECM dynamics, and these results give insight into additional ECM alterations in UCPPS.

The remaining validated UCPPS: HC proteins are predominantly related to host defense and immune signaling. CD99 is a ubiquitously expressed multifunctional protein (22) that is critical to leukocyte (23), monocyte (24), neutrophil (25), and t-cell (26) transmigration, among other functions. The decreased CD99 may reflect decreased CD99 in bladder tissues that would cause impaired migration of these immune cells. TNFRSF12A (also known as FGF-inducible molecule 14) is the receptor for TNF-related weak inducer of apoptosis, which is produced in leukocytes. TNFRSF12A is important to maintaining tissue homeostasis. Tissue injury, oxidative stress, and inflammatory responses increase TNF-related weak inducer of apoptosis, activating TNFRSF12A, and if done in excess can cause fibrosis (27, 28) and tissue damage. However, more moderate TNFRSF12A signaling is important for wound repair in a variety of tissue injury models (29, 30, 31). The decrease of TNFRSF12A in UCPPS may underlie an insufficient wound healing response in UCPPS. DEF4 has antibiotic and cytotoxic activity and is part of the host defense system in the bladder. Its relative decrease in UCPPS may reflect a loss of a critical host defense factor. The related defensin, DEF3, is also decreased in the full UCPPS:HC comparison. Having determined that levels of these specific urinary proteins are altered in UCPPS, future studies could use tailored approaches, such as ELISA for specific proteins, in larger cohorts to define the diagnostic potential of these quantitative changes.

The secondary objective of this study was to leverage the ability of unbiased proteomics to identify dysregulated biological functions, networks, and upstream regulators associated with UCPPS. Although validation is a critical part of any proteomic study, publication of the full set of proteins implicated in the full cohort of UCPPS is also critical to the prediction of altered regulators and networks. In this context, maximizing the sensitivity of the analysis can be critical to determining relevant biological processes and regulators of the disease. UCPPS is poorly understood and identification of putative regulators will enable the generation of novel hypotheses and therapeutic targets to evaluate in future studies.

Males and females have broadly shared UCPPS phenotypic characteristics of pain and urinary frequency and urgency. In this study, we have identified key proteomic differences between the male and female UCPPS: HC differentially expressed proteins. These are numerous, and the complete results are listed in supplemental data 1. Some particularly intriguing sex-specific differences include a statistically significant, 1.9-fold increase in bifunctional heparan sulfate N-deacetylase/N-sulfotransferase 1 exclusively in females with UCPPS. N-deacetylase/N-sulfotransferase 1 is required for the synthesis of heparin sulfate, creating ligands that can interact with L-selectin and mediate leukocyte migration. The most significant UCPPS: HC protein among females is GLG1. GLG1 is upregulated 1.6-fold and binds E-selectin, meaning that two of the most significant increased female UCPPS: HC proteins influence or directly bind selectins. Altered levels of these two proteins likely affect the empirical observations over the years of increased leukocytes in the urine of UCPPS. Neither of these proteins were statistically significant in males, perhaps suggesting a potential sex-specific modulation of selectin signaling in females. Future studies must also consider sex-associated differences in healthy controls in the context of any changes in UCPPS as several urinary proteins are different in healthy males and females.

A comparative analysis of UCPPS and the diverse diseases comprising PC in this study cohort suggests that UCPPS is uniquely characterized among them as having impaired cell movement and leukocyte migration. Placing these findings in a complete biological context requires a comparative analysis of all three of UCPPS, PC, and HC however. The identification of UCPPS-specific regulators that drive the disease phenotype is a critical goal for UCPPS research. In this study, several UCPPS-specific regulators were predicted based on the quantitative urinary profiles obtained. Alpha-catenin signaling was predicted to be increased, with a z-score of 2.2 in the UCPPS: HC comparison. Alpha-catenin affects cell morphogenesis, cell cohesion, and alters intracellular actin dynamics. The decreases were predicted for UCPPS for the important and multifunctional growth factors TGFB1 and epidermal growth factor, possibly reflecting a decreased epithelial regenerative capacity. TGFB1 also affects inflammation, immunity, wound repair, and other processes.

Although prior urinary proteomic studies have not been performed, other UCPPS studies display several similar findings supporting our conclusions. Histological studies have long described increased immune cell infiltrates in bladder biopsy samples, increased vasculogenesis, alterations in ECM as evidenced by denudation of bladder epithelium and altered collagen levels, and elevated inflammation; particularly so among those subjects with verified Hunner's ulcers (32, 33, 34, 35, 36). It is notable that we also can identify these same pathways noninvasively by urinary proteomics. More recently, transcriptomic profiling of bladder tissue (37) highlighted elevated RNA associated with immune system regulation, inflammation, and immune cell chemotaxis, similar to both older reports (38, 39, 40) and our urinary proteomic data presented in this study.

As with any study on clinical samples, obtaining a full and complete characterization of each individual in the study was not possible. In particular, although extensive efforts were undertaken to phenotype these patient cohorts, a complete characterization of the therapeutic profile could not be undertaken. The influence of antibiotics and/or other drug therapies cannot be completely ruled out. Importantly, there were no indications of active urinary tract infection on urine dipstick analysis, and UCPPS subjects are repeatedly assessed for urinary tract infections.

This study is the first unbiased proteomic report of UCPPS patient urine. These data also provide support for prior hypotheses that a compromised barrier function is important to the etiology of UCPPS, particularly in females. In addition, this study identifies novel urinary proteins that could be influencing disease and symptom progression and implicates upstream regulators that, after further study and verification, could potentially be exploited therapeutically and diagnostically.

## Data availability

The mass spectrometry proteomics data have been deposited to the ProteomeXchange Consortium *via* the PRIDE partner repository with the dataset identifier PXD025005.

## Supplemental data

This article contains supplemental data.

## Conflict of interest

The authors declare no competing interests.

## Supplemental data

Supplemental data 1: Excel file detailing additional analyses.

Supplemental data 2: List of identified peptides.

Supplemental data 3: List of master proteins.

Supplemental data 4: List of all proteins, with per-file figures of merit.

Supplemental data 5: Peptide-level quantification.

Supplemental data 6: Protein group IDs for master proteins.

Supplemental data 7: Protein FDR and quant on a per-file basis.
